# Mechanisms Responsible for the Compositional Heterogeneity of Nascent High Density Lipoprotein*

**DOI:** 10.1074/jbc.M113.495523

**Published:** 2013-07-08

**Authors:** Sissel Lund-Katz, Nicholas N. Lyssenko, Margaret Nickel, David Nguyen, Palaniappan Sevugan Chetty, Ginny Weibel, Michael C. Phillips

**Affiliations:** From the Lipid Research Group, The Children's Hospital of Philadelphia, The University of Pennsylvania Perelman School of Medicine, Philadelphia, Pennsylvania 19104-4318

**Keywords:** ABC Transporter, Apolipoproteins, Cholesterol, High Density Lipoprotein (HDL), Lipid Raft, Lipoprotein Structure, Membrane Lipids, Phospholipid, Sphingolipid, Phospholipids

## Abstract

Apolipoprotein (apo) A-I-containing nascent HDL particles produced by the ATP binding cassette transporter A1 have different sizes and compositions. To understand the molecular basis for this heterogeneity, the HDL particles produced by apoA-I-mediated solubilization of phospholipid (PL)/free (unesterified) cholesterol (FC) bilayer membranes in cell and cell-free systems are compared. Incubation of apoA-I with ATP binding cassette transporter A1-expressing baby hamster kidney cells leads to formation of two populations of FC-containing discoidal nascent HDL particles. The larger 11-nm diameter particles are highly FC-enriched (FC/PL = 1.2/1 mol/mol) relative to the smaller 8 nm particles and the cell plasma membrane (FC/PL = 0.4/1). ApoA-I-mediated spontaneous solubilization of either multilamellar or unilamellar vesicles made of a membrane-PL mixture and FC yields discoidal HDL particles with diameters in the range 9–17 nm and, as found with the cell system, the larger particles are relatively enriched in FC despite the fact that all particles are created by solubilization of a common FC/PL membrane domain. The size-dependent distribution of FC among HDL particles is due to varying amounts of PL being sequestered in a boundary layer by interaction with apoA-I at the disc edge. The presence of a relatively large boundary layer in smaller discoidal HDL promotes preferential distribution of phosphatidylserine to such particles. However, phosphatidylcholine and sphingomyelin which are the primary PL constituents of nascent HDL do not exhibit selective incorporation into HDL discs of different sizes. This understanding of the mechanisms responsible for the heterogeneity in lipid composition of nascent HDL particles may provide a basis for selecting subspecies with preferred cardio-protective properties.

## Teaser

**Background:** The molecular basis for heterogeneity in nascent HDL particle cholesterol content is unknown.

**Results:** Apolipoprotein A-I-mediated solubilization of phospholipid/cholesterol membranes creates large cholesterol-rich and small cholesterol-poor HDL particles.

**Conclusion:** Variations in cholesterol distribution between nanoscale HDL discs are due to particle size-dependent molecular packing constraints.

**Significance:** This explains the origin of HDL particles with different cholesterol contents.

## Introduction

Plasma HDL comprises a mixture of different particles, and this heterogeneity is significant because the various subspecies exhibit different biological activities (1, 2). It is important to understand the factors responsible for this heterogeneity because such knowledge may provide the capability to manipulate the distribution of HDL subspecies and thereby HDL functionality. The heterogeneity of HDL arises during the biogenesis process because nascent particles with different compositions are created simultaneously (3–5). Formation of these different HDL particles occurs before there is remodeling by plasma factors such as lipid transfer proteins and lipases. In the case of apolipoprotein (apo)^2^ A-I-containing particles, nascent HDL particles are created by apoA-I-mediated solubilization of cell plasma membrane domains formed by the activity of the ATP binding cassette transporter AI (ABCA1) (6). The rate-limiting step in this process is the solubilization of vesiculated domains of phospholipid (PL)/cholesterol bilayer membrane by apoA-I to create discoidal HDL particles of different sizes and compositions. We have elucidated some of the factors that control the heterogeneity of these nascent HDL particles (7) but the mechanisms responsible for variations in cholesterol content and PL class distribution remain unresolved. For instance, it is not clear whether the presence of ABCA1 in different membrane microenvironments influences the lipid composition of nascent HDL particles (3, 4, 8, 9).

Here, we exploit the fact that the factors controlling HDL particle heterogeneity are the same in cell systems where ABCA1 is active and in cell-free systems where PL vesicles are incubated directly with apoA-I (7). This correspondence occurs because the process of PL solubilization by apoA-I controls HDL particle formation in both systems. We employ model lipid mixtures to discover how cholesterol and different classes of PL distribute to reconstituted HDL particles of different sizes. The information gained from such experiments helps explain the lipid compositions of nascent HDL particles created when apoA-I is incubated with ABCA1-expressing cells. Solubilization of macroscopically homogeneous PL/cholesterol membranes by apoA-I can give rise to discoidal HDL particles that are heterogeneous with respect to cholesterol/PL ratio and PL class distribution. Knowledge of the mechanisms responsible for the variability in nascent HDL lipid composition may lead to ways of controlling the population of HDL subspecies formed, thereby allowing selection of the most functionally desirable subspecies.

## EXPERIMENTAL PROCEDURES

### 

#### 

##### Materials

ApoA-I was isolated from human HDL and radiolabeled to a specific activity of ∼1 μCi/mg by reductive methylation using either ^3^H- or ^14^C-labeled formaldehyde (American Radiolabeled Chemicals, Inc.) as described previously (3). The following lipids were purchased from Avanti Polar Lipids: free (unesterified) cholesterol (FC), egg sphingomyelin (SM), pig brain SM, dipalmitoyl phosphatidylcholine (DPPC), dimyristoyl phosphatidylcholine (DMPC), pig brain phosphatidylserine (PS), and egg lyso-PC. [^3^H]cholesterol (51 Ci/mmol) and [^14^C]cholesterol (50 mCi/mmol) were obtained from Perkin Elmer Life Sciences. [^14^C]Palmitoyl SM (50–60 mCi/mmol), [^14^C]dioleoyl PS (50–60 mCi/mmol) and [^3^H]DPPC (30–60 Ci/mmol) were purchased from American Radiolabeled Chemicals, Inc.

##### Cell Culture

Baby hamster kidney (BHK) cells expressing human ABCA1 (BHK-ABCA1) under the control of a mifepristone-inducible promoter (10) were maintained in Dulbecco's modified Eagle's medium containing 10% fetal bovine serum and 50 μg/ml gentamicin at 37 °C in 5% CO_2_ (7, 11). Cells were treated with 10 nm mifepristone to express ABCA1 and incubated with apoA-I (10 μg/ml) to induce lipid efflux and formation of nascent HDL particles (7, 12). Plasma membrane vesicles were produced by incubating the cells with 50 mm formaldehyde and 2 mm dithiothreitol for 90 min (13). Detergent-free plasma membrane lipid raft and non-raft domains were obtained from cell lysates by flotation in an OptiPrep gradient (14).

##### Isolation of Nascent HDL

Conditioned medium from the BHK-ABCA1 cells described above was concentrated 20-fold using an Amicon Ultracel-10K centrifugal filter and fractionated by gel filtration chromatography on a calibrated HiLoad 16/60 Superdex 200 column using an Akta FPLC system, as described previously (3, 4, 7). The particle sizes of the various nascent HDL fractions were determined by comparing their *K*_av_ values with those of proteins of known hydrodynamic diameter.

##### Reconstituted HDL

The kinetics of solubilization of PL MLV by apoA-I to form discoidal apoA-I·PL complexes were measured by monitoring the decreases in absorbance at 325 nm, as described before (6, 15). MLV containing a DMPC/FC (3 weight %) mixture were incubated with apoA-I at a 2:1 (w/w) lipid/apoA-I ratio at 26 °C (16). MLV formed from a membrane PL mixture (37.5% egg SM, 37.5% DPPC, 20% brain PS, and 5% egg lyso-PC) containing 5 weight % FC were prepared by dispersing a dry film of the lipid mixture in TBS using vortexing and storing the suspension overnight at 4 °C. The MLV were then incubated at 37 °C for 30 min and then mixed with an apoA-I solution in TBS at the same temperature to give a 1:1 (w/w) lipid/apoA-I ratio. The mixture was then incubated at 37 °C with gentle shaking for 24 h. Reconstituted HDL particles were also prepared from these mixtures using the cholate dialysis method (17) as described previously (18). To monitor the distributions of the various constituents among the HDL products formed in the above solubilization reactions, the initial MLV mixtures were variously trace-labeled with either [^3^H] or [^14^C]cholesterol, PL, and apoA-I. The HDL particles of different sizes were separated by FPLC and the distribution of the radiolabels between the fractions was determined using liquid scintillation counting (7). All experiments of this type were repeated at least twice using a fresh preparation of MLV in each case. The reconstituted HDL particles were examined by negative staining electron microscopy as described (4). Chemical cross-linking was employed to assess the number of apoA-I molecules per HDL particle (4, 7, 19). The α-helix content of the apoA-I was determined from CD spectra measured with a Jasco 810 spectropolarimeter using the molar ellipticity at 222 nm as described previously (20).

##### Analytical Procedures

The protein contents of the HDL preparations were determined using a modified Lowry method (21). The HDL lipids were extracted by the Bligh-Dyer procedure (22). A gas-liquid chromatographic method (23) was employed to assay the cholesterol, and the PL content was determined by assaying for inorganic phosphorus (24). The concentration of choline-PL was determined using the phospholipid C kit (Wako Chemicals). An HPLC analysis was used to separate and determine the amounts of different classes of PL (25).

## RESULTS

### 

#### 

##### Nascent HDL Produced by BHK-ABCA1 Cells

**FIGURE 1.**
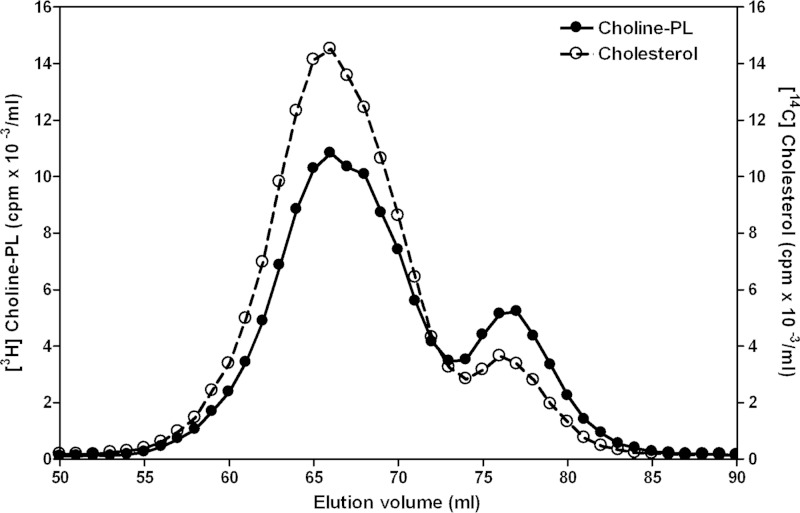
**Gel filtration elution profiles of apoA-I-containing nascent HDL particles formed by BHK-ABCA1 cells.** [^14^C]cholesterol- and [^3^H]choline-labeled cells were treated overnight with 10 nm mifepristone and then incubated with 20 μg/ml apoA-I for 8 h. The conditioned medium was analyzed as described in Methods. The distributions of [^3^H] choline-PL (●) and [^14^C]cholesterol (○) radioactivity reflect formation of two different sizes of nascent HDL particles. The population of larger particles elutes with a peak maximum near 65 ml, which corresponds to a hydrodynamic diameter of ∼11 nm and the population of smaller particles elutes near 77 ml and has a diameter of ∼8 nm.

Recently, we have used BHK-ABCA1 cells to show that the ratio of cell lipid made available as a consequence of ABCA1 activity to apoA-I in the extracellular medium is the fundamental parameter controlling nascent HDL size heterogeneity (7). The populations of large and small HDL particles that form in such cell-based systems arise in an analogous fashion when discoidal apoA-I-containing HDL particles are reconstituted in cell-free systems. The present experiments are designed to reveal factors that determine the lipid compositions of different sizes of nascent HDL particles. Fig. 1 shows how cholesterol and choline-PL released from BHK-ABCA1 cells to extracellular apoA-I distributes between the populations of large (∼ 11-nm hydrodynamic diameter) and small (∼ 8-nm diameter) nascent HDL particles that are formed (7). It is apparent that the ratio of FC to PL is higher in the larger HDL particles, as has been observed with other cell types (3–4, 9, 26). Mass analysis of the isolated nascent HDL particles indicates that the FC/PL ratio is approximately three times greater for the large particles (Table 1); cholesteryl ester could not be detected in the nascent HDL particles (a level of 1% relative to FC would have been apparent). Relative to PL, the mol % FC in the large nascent HDL is 55% as compared with 30% for both small nascent HDL and the average value for the plasma membrane. These FC-rich HDL particles are disc-shaped because they form characteristic rouleaux when examined by negative stain electron microscopy (data not shown). Table 1 also summarizes the classes of PL that are incorporated into large and small nascent HDL particles. Relative to the BHK-ABCA1 cell plasma membrane, the content of acidic PL (PS + PE) is reduced in both sizes of nascent HDL. It is well established that the predominant classes of PL contained in nascent HDL are PC and SM, although the PC/SM ratio and the degrees of incorporation of the minor classes of PL vary with cell type (*cf.* Refs. 3, 4, 9, 27–36).

**TABLE 1:** **Lipid compositions of apoA-I-containing nascent HDL particles formed by BHK-ABCA1 cells** Cells were grown and nascent HDL isolated as described in Fig. 1 (20 μg/ml apoA-I, 10 nm mifepristone).

Component	FC/PL (w/w)	PL composition (% w/w)			
		PC	SM	Lyso-PC	Acidic PL
Large nascent HDL	0.61 ± 0.05 (*n* = 7)	65 ± 3*^a^* (*n* = 9)	20 ± 5*^b^*	4 ± 2	10 ± 3
Small nascent HDL	0.21 ± 0.03 (*n* = 3)	51 ± 2*^a^* (*n* = 3)	30 ± 6*^b^*	5 ± 2	13 ± 2*^c^*
Plasma membrane vesicles*^d^*	0.21 ± 0.03 (*n* = 3)	49 ± 2	27 ± 1	4 ± 2	20 ± 2*^c^*

*^a^* Values are significantly different (*p* = 0.02) by unpaired *t* test.

*^b^* Values are significantly different (*p* = 0.02) by unpaired *t* test.

*^c^* Values are significantly different (*p* = 0.01) by unpaired *t* test.

*^d^* Plasma membrane vesicles were isolated as described under “Experimental Procedures” from BHK cells exposed to 10 nm mifepristone.

**FIGURE 2.**
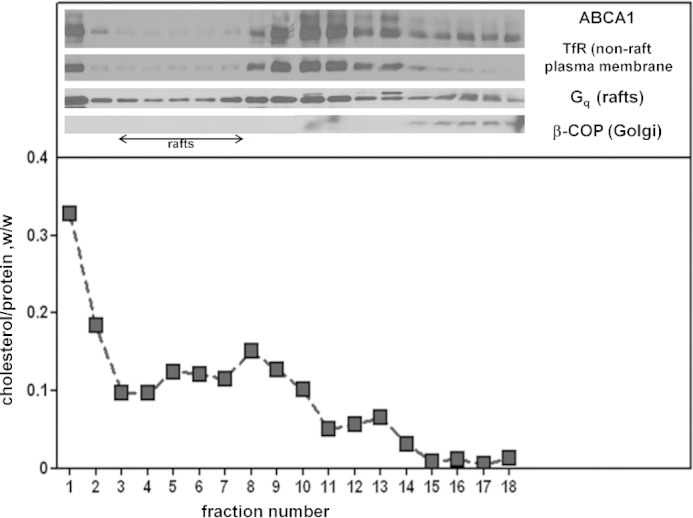
**Analysis of the distribution of ABCA1 between different membrane domains.** Detergent-free lipid rafts were isolated from lysed BHK-ABCA1 cells as described under “Experimental Procedures.” The *top panel* shows the distribution of ABCA1 and some marker proteins across the OptiPrep gradient. An aliquot of each gradient fraction was analyzed by SDS-PAGE followed by Western blotting for the indicated protein. *TfR*, transferrin receptor; *G_q_*, G protein subunit. The *lower panel* shows the relative distribution of cholesterol and protein across the OptiPrep gradient.

The observed FC and PL composition of nascent HDL has led to the suggestion by us and others that lipid-raft domains in the plasma membrane may be the source of lipids, especially for larger nascent HDL particles (4, 9). This concept seems reasonable if ABCA1 located in lipid-raft domains of the plasma membrane actively reorganizes them (37, 38) making the lipids therein more accessible to apoA-I. ABCA1 can apparently distribute between lipid-raft and non-raft domains of the plasma membrane with the distribution being dependent on cell type (38, 39). However, the preferred location for ABCA1 is the more fluid non-raft regions of the plasma membrane (8, 37, 38) and the data in Fig. 2 showing that the transporter in BHK-ABCA1 cells is located in non-lipid raft domains is consistent with this concept. Consequently, the nascent HDL lipid compositions summarized in Table 1 are the result of the activity of ABCA1 located only in non-lipid raft domains of the plasma membrane. This observation raises the question of how heterogeneous nascent HDL lipid compositions arise if ABCA1 is not located in variable membrane microenvironments. The question is particularly pertinent with respect to the enrichment of FC in large nascent HDL relative to the plasma membrane (Table 1), especially in light of recent direct chemical evidence that the SM domains that exist in the plasma membrane due to the influence of the cytoskeleton (40) are not enriched in cholesterol and that cholesterol is uniformly distributed throughout the plasma membrane (41). To better understand the lipid heterogeneity between large and small nascent HDL, we take advantage of the fact that the mechanism of nascent HDL particle formation involves solubilization of vesiculated PL in both cell and cell-free systems (6, 7).

##### HDL Particles Reconstituted from a Lipid Membrane Mixture

**FIGURE 3.**
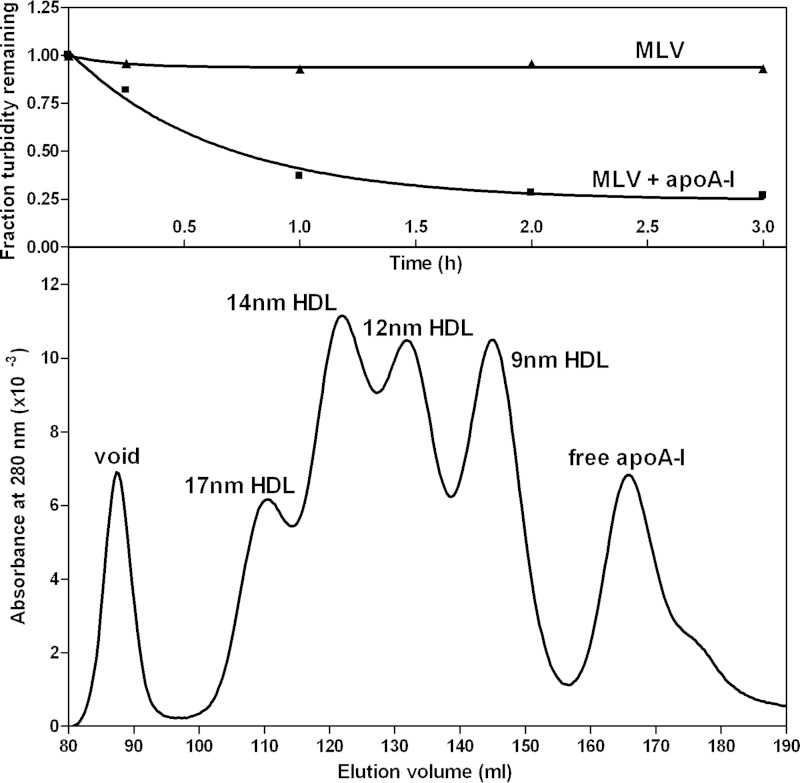
**Solubilization of membrane-lipid MLV by apoA-I.** The membrane lipid mixture comprised 37.5% (w/w) egg SM, 37.5% DPPC, 20% brain PS, and 5% egg lyso-PC to which 5% by weight cholesterol was added. The MLV were incubated with apoA-I (1 mg of lipid + 1 mg of apoA-I in 2 ml of TBS) at 37 °C with gentle shaking. The *top panel* shows a representative time course (■) for clearance of MLV by apoA-I. The MLV were also incubated in the absence of added apoA-I (▴). The *bottom panel* shows the gel filtration elution profile of an MLV/apoA-I mixture after incubation for 24 h at 37 °C. The protein distribution (as monitored by absorbance at 280 nm) indicates that HDL particles with hydrodynamic diameters of 17, 14, 12, and 9 nm are formed (designated HDL peaks 1–4 in Table 2).

**FIGURE 4.**
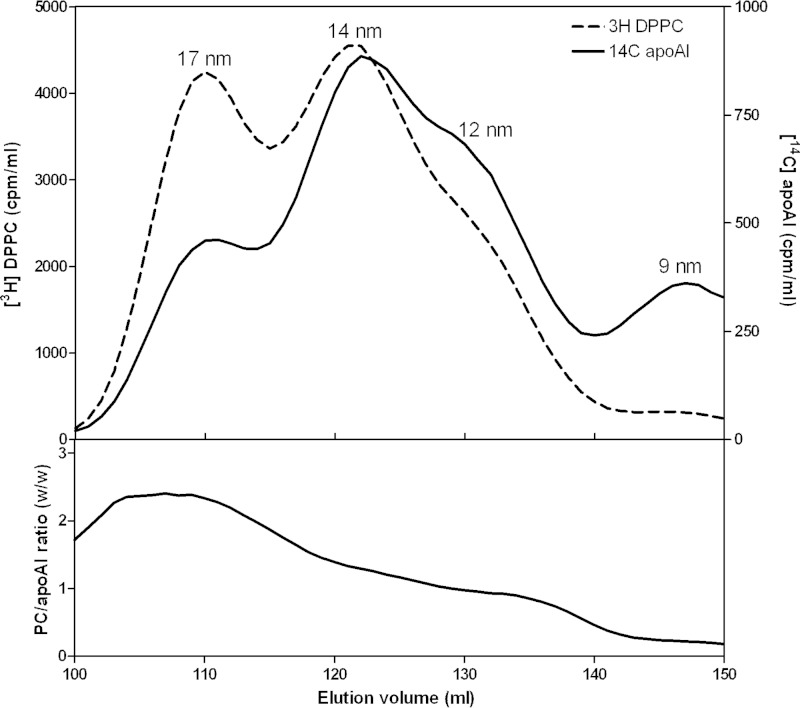
**Relative distributions of DPPC and apoA-I between the four sizes of HDL particles formed by solubilization of membrane-lipid MLV by apoA-I at 37 °C.** The MLV contained [^3^H]DPPC and were incubated with [^14^C]apoA-I, and the HDL particles formed were fractionated by gel filtration chromatography as described in the legend to Fig. 3. The *top panel* shows the distributions of [^3^H]DPPC and [^14^C]apoA-I radioactivity across the elution profile. The *bottom panel* shows the DPPC/apoA-I mass ratio (calculated from the specific radioactivities) across the elution profile.

To achieve the goal of understanding the molecular mechanisms underlying the heterogeneity in lipid composition of nascent HDL particles, it was necessary to develop a vesicle of defined PL and FC composition that forms a range of HDL particles of different sizes when solubilized by apoA-I. Fig. 3 describes such a “membrane-lipid” mixture containing the major PL classes found in the plasma membrane of mammalian cells. MLV containing this representative membrane lipid mixture are spontaneously converted by reaction with apoA-I at 37 °C into discoidal HDL particles with diameters in the range 9–17 nm (Fig. 3). The characteristics of some of these particles are listed in Table 2. As expected, the larger discs exhibit a higher PL/apoA-I ratio. Fig. 4 shows an explicit comparison of the relative distributions of DPPC and apoA-I across the four sizes of discoidal HDL particles formed when the MLV are solubilized by apoA-I. The results are consistent with those in Table 2 in showing that the larger HDL particles contain more PC per apoA-I molecule; this finding is consistent with prior literature on reconstituted HDL particles formed from a single type of PL (16, 42, 43).

**TABLE 2:** **Properties of apoA-I-containing HDL particles formed by solubilization of “membrane PL” MLV at 37 °C** The MLV were mixed at a 1:1 (w/w) total lipid/apoA-I ratio and incubated as described under “Experimental Procedures.”

Peak number*^a^*	HDL particle diameter (nm)		ApoA-I mol/particle*^d^*	α-Helix content*^e^*	Choline PL/apoA-I*^f^*	FC/apoA-I*^f^*
	Gel filtration*^b^*	em*^c^*				
				%	*mol/mol*	*mol/mol*
2–3	12–14	20 ± 1	2	68	48 ± 12	6 ± 2
4	9	18 ± 1	2	53	10 ± 2	0.4 ± 0.2

*^a^* The four HDL peaks are identified in the gel filtration profile shown in Fig. 3. Peak 1 (17 nm HDL) was not analyzed because of limited material and peaks 2 and 3 were analyzed together because of incomplete resolution.

*^b^* The gel filtration column was calibrated as described under “Experimental Procedures” to give hydrodynamic diameters.

*^c^* The major diameters (mean ± S.D.) of the discoidal particles were measured from negative stain electron micrographs using the program “Gatan Digital Micrograph” and counting at least 100 particles appearing as rouleaux.

*^d^* The number of apoA-I molecules was determined using chemical cross-linking and SDS-PAGE.

*^e^* The helix content (accurate to within 5%) was derived from CD spectra, as described under “Experimental Procedures.”

*^f^* The particle lipid contents are presented as mean ± S.D. (*n* = 4).

##### FC Distribution between HDL Particles of Different Sizes

**FIGURE 5.**
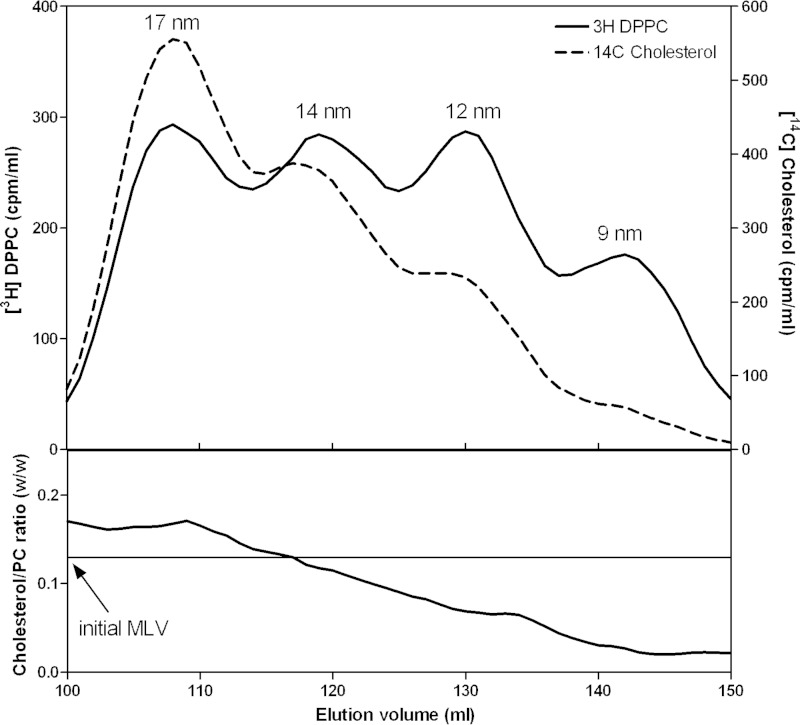
**Relative distributions of DPPC and cholesterol between the four sizes of HDL particles formed by solubilization of membrane-lipid MLV by apoA-I at 37 °C.** The MLV contained [^3^H]DPPC and [^14^C]cholesterol. The *top panel* shows the distributions of the two radiolabels across the elution profile. The *lower panel* shows the cholesterol/DPPC mass ratio across the elution profile. The *horizontal line* shows the cholesterol/DPPC ratio present in the initial MLV.

The mass analysis in Table 2 shows that the FC/apoA-I ratio for the 12–14 nm discoidal HDL particles reconstituted from the membrane-lipid MLV is larger than the ratio for the 9 nm disc. This finding is consistent with the observation that the larger nascent HDL particles obtained with the BHK-ABCA1 cells are relatively FC-rich (Table 1). Fig. 5 presents a detailed comparison of the relative distributions of FC and DPPC between the four sizes of discoidal HDL particles formed in the apoA-I-mediated MLV solubilization reaction. Inspection of the distributions of the FC and DPPC radiolabels reveals that the 17-nm HDL particles are relatively enriched in FC compared with their smaller counterparts. The data in the *lower panel* of Fig. 5 show how the FC/PC mass ratio decreases by a factor of ∼8 between the 17-nm and 9-nm HDL particles. It should be noted that the 17-nm HDL particles are also enriched in FC compared with the initial MLV. Importantly, none of the four sizes of HDL particles contains the same ratio of FC/PC as was present in the initial MLV. This observation indicates that the FC content of the HDL particles is not simply determined by the level of FC in the membrane that is solubilized by apoA-I. In the case of MLV, apoA-I interacts with the surface of particles with diameters in the micrometer range. To determine whether reducing the size of the domain of FC/PL bilayer membrane that apoA-I interacts with has any influence on HDL particle formation and composition, the MLV experiment summarized in Fig. 5 was repeated using 20-nm small unilamellar vesicles (SUV) of the same composition. The SUV were created by sonicating the MLV before addition of apoA-I to induce solubilization and formation of discoidal HDL particles. The same four sizes of HDL particles were created, and the FC/PC mass ratio was similar to that shown in the *lower panel* of Fig. 5 (data not shown). The 17-nm HDL particles were relatively enriched in FC relative to the initial SUV.

**FIGURE 6.**
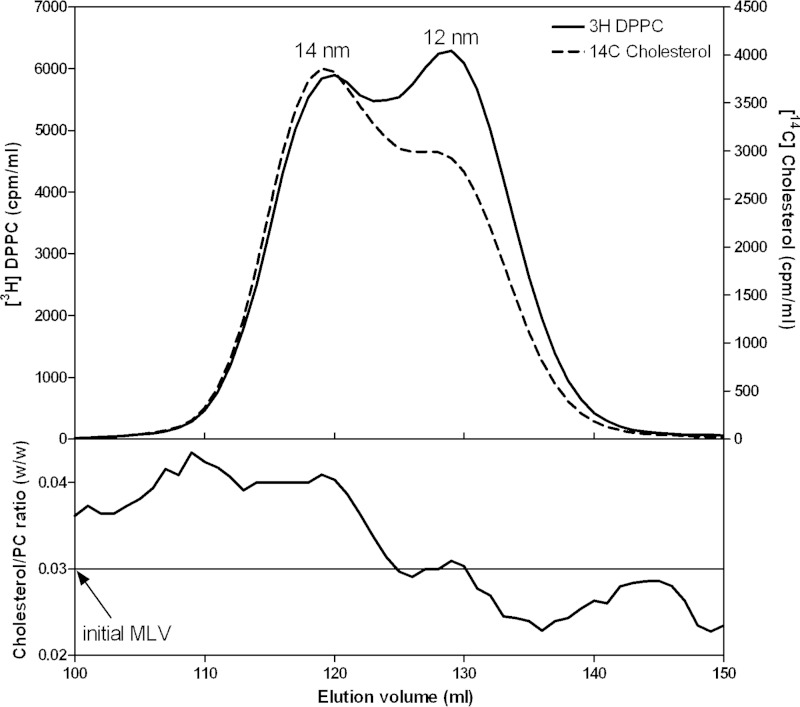
**Relative distributions of DMPC and cholesterol between the two populations of HDL particles formed when DMPC/FC (97/3 w/w) MLV are solubilized by apoA-I at 26 °C (see “Experimental Procedures”).** The DMPC MLV contained a measured trace of [^3^H]DPPC to monitor the DMPC distribution and [^14^C]cholesterol and were incubated with apoA-I as described under “Experimental Procedures.” The two sizes of HDL particles formed were fractionated by gel filtration chromatography. The *top panel* shows the distributions of [^3^H]DPPC marker and [^14^C]cholesterol across the elution profile. The *lower panel* shows the FC/DMPC mass ratio across the elution profile.

Because the MLV used in the experiment described in Fig. 5 are formed from a membrane PL mixture (PC+SM+PS+lyso-PC), it is possible that the variations in FC/PL mass ratio between the four sizes of HDL particles are a consequence of different distributions of the classes of PL between these HDL particles. To eliminate any confounding contributions of this effect, MLV formed from DMPC and cholesterol were utilized. The ability of apoA-I to solubilize such MLV and create discoidal HDL particles has been characterized in detail (16, 44–47). Conditions described by Massey and Pownall (16) were employed in the experiment described in Fig. 6. In this case, 12- and 14-nm HDL particles are formed and the distributions of PC and FC radiolabels (*top panel* in Fig. 6) indicate that the FC/PC ratio is higher in the larger particles (*cf.* Ref. 16). When the same experiment is conducted using [^14^C]cholesterol and [^3^H]apoA-I, the FC/apoA-I ratio is higher for the 14-nm HDL particle than for the 12-nm particle (data not shown). The data in the *lower panel* of Fig. 6 show that the FC/PC mass ratio is higher for the 14-nm HDL particle relative to the ratio for both the 12-nm particle and the initial MLV. Thus, the FC/PL distribution data in Figs. 5 and 6 indicate that the preferential incorporation of cholesterol into larger discoidal HDL particles is a consequence of their size alone and not differences in the type of PL present.

##### Distributions of Different Classes of PL between HDL Particles of Different Sizes

**FIGURE 7.**
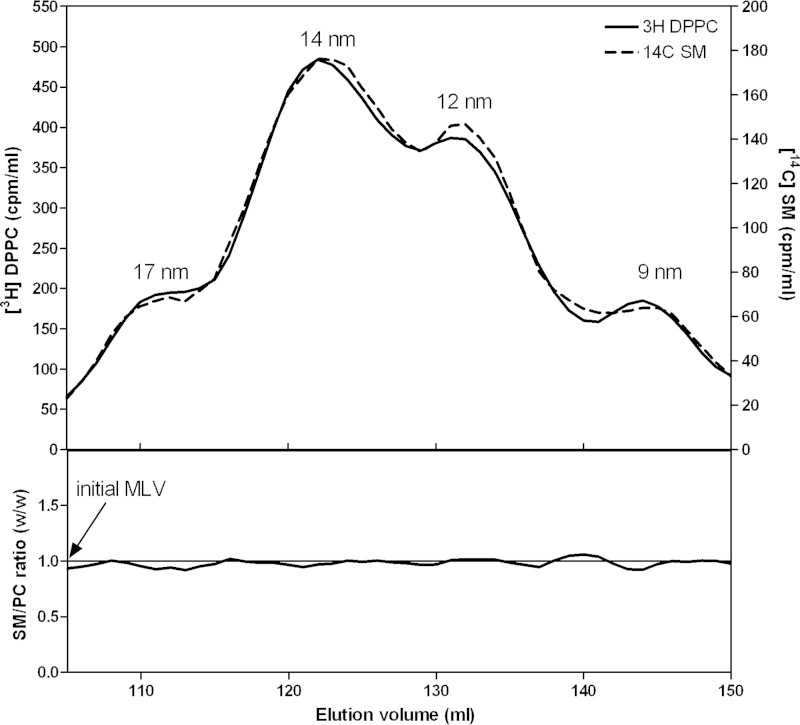
**Relative distributions of DPPC and SM between the four sizes of HDL particles formed by solubilization of membrane-lipid MLV by apoA-I at 37 °C.** The MLV contained [^3^H]DPPC and [^14^C]SM. The *top panel* shows the distributions of the two radiolabels across the elution profile. The *lower panel* shows the SM/DPPC mass ratio across the elution profile. The *horizontal line* shows the SM/DPPC ratio present in the initial MLV.

**FIGURE 8.**
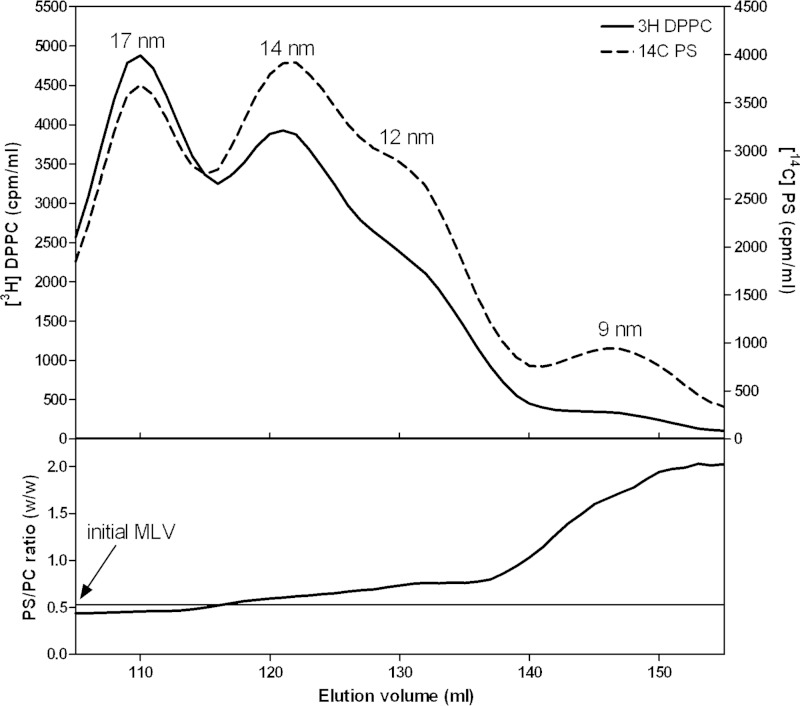
**Relative distributions of DPPC and PS between the four sizes of HDL particles formed by solubilization of membrane-lipid MLV by apoA-I at 37 °C.** The MLV contained [^3^H]DPPC and [^14^C]PS. The *top panel* shows the distributions of the two radiolabels across the elution profile. The *lower panel* shows the PS/DPPC mass ratio across the elution profile. The *horizontal line* shows the PS/DPPC ratio present in the initial MLV.

The distributions of PL classes between nascent HDL particles of different sizes can vary, as exemplified by the data in Table 1 for the HDL particles formed with BHK-ABCA1 cells. It is important to know whether such variations in PL composition of HDL can arise when a membrane of given composition is solubilized by apoA-I. We used the membrane-PL MLV solubilization assay summarized in Fig. 3 to address this issue. The relative distributions of PC, SM, and PS between the four sizes of HDL particles created when such MLV are solubilized by apoA-I at 37 °C were determined using the appropriately radiolabeled PL. Fig. 7 shows that DPPC and egg SM co-distribute among the various HDL; this result is consistent with the binary phase diagram for these PL showing that they are completely miscible in bilayers at 37 °C (48). An equivalent experiment is shown in Fig. 8 and, in this case, the two types of PL distribute unequally among the various sizes of HDL particles. The larger 17-nm particles contain a ratio of PS/PC very similar to that in the initial MLV, but this ratio increases markedly for the 9-nm HDL particle. This preferential distribution of PS into the smaller HDL was confirmed by isolating the PL from such particles and performing an HPLC analysis (data not shown).

##### Influence of PL Acyl Chain Composition on HDL Size Distribution

**FIGURE 9.**
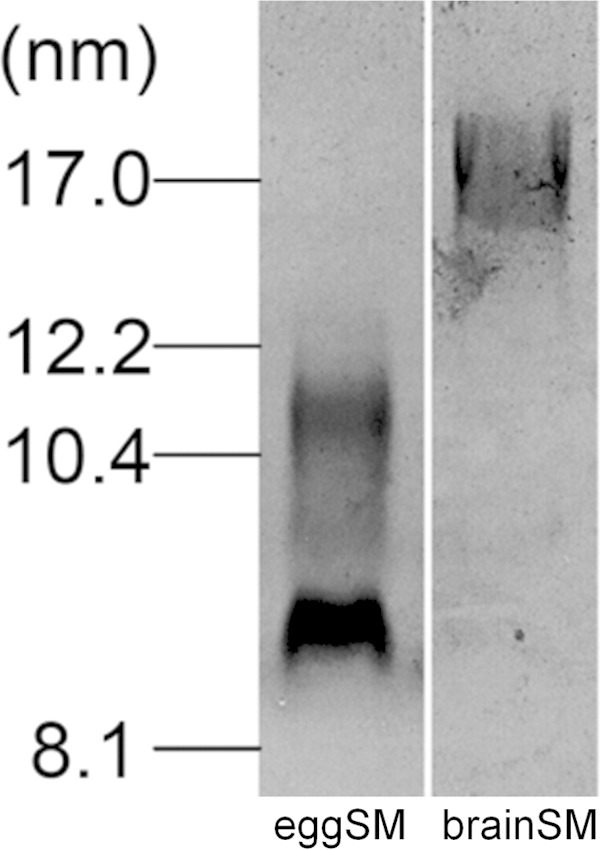
**Sensitivity of the size of HDL particles formed by membrane PL solubilization to PL acyl chain composition.** SM MLV were solubilized with apoA-I by incubation at 37 °C (2.5:1 (w/w) SM/apoA-I) and the sizes of the resultant HDL particles were determined using native 4–12% gradient PAGE and staining with Coomassie Blue. The predominant *N*-acylation of the egg SM was 84% 16:0. The predominant *N*-acylation of the pig brain SM was 45% 18:0 and 30% 22:0 and 24:0.

It is known that different distributions of particle sizes arise when HDL particles are reconstituted by the cholate dialysis method with PC molecules containing different acyl chains (49). The data in Fig. 9 show that reconstituted HDL particle size is also extremely sensitive to PL acyl chain composition when MLV are directly solubilized by apoA-I at 37 °C. It is apparent that whereas egg SM, which possesses 16:0 *N*-acylation, forms 9- and 11-nm diameter HDL particles, pig brain SM, which contains longer saturated *N*-acyl chains, forms markedly larger 17-nm HDL particles. It follows that relatively subtle alterations in PL acyl chain composition can have marked effects upon the sizes of HDL particles created by apoA-I-mediated spontaneous solubilization.

## DISCUSSION

The discoidal nascent HDL particles created by BHK-ABCA1 cells are typical in that they are heterogeneous in size with larger particles being relatively lipid-rich. In particular, particles in the larger 11-nm diameter population contain more than equimolar amounts of FC relative to PL, which is greater than the ratio occurring in the plasma membrane of the BHK cells. As is generally observed with nascent HDL particles, PC and SM are the predominant PL species with some variability in the relative amounts of these two species occurring. In both cell and cell-free systems, the ratio of available lipid/apoA-I is a major determinant of the size distribution of the discoidal HDL particles created (7). An unresolved question is how does apoA-I-mediated solubilization of plasma membrane lipids create discoidal HDL particles with different FC contents? As mentioned earlier, this phenomenon is not a consequence of ABCA1 being located in plasma membrane raft and non-raft domains that contain different levels of FC (Fig. 2). Because apoA-I-mediated solubilization of either PL/FC MLV or SUV, which have a common FC content, yields discoidal HDL particles with FC contents that vary similarly (Fig. 5), it follows that this heterogeneity in composition arises during solubilization of a vesiculated membrane domain that is uniform down to the scale of 20 nm (the diameter of a SUV). The mechanism responsible for this effect is considered in the following section.

### 

#### 

##### Influence of HDL Disc Size on FC Content

**FIGURE 10.**
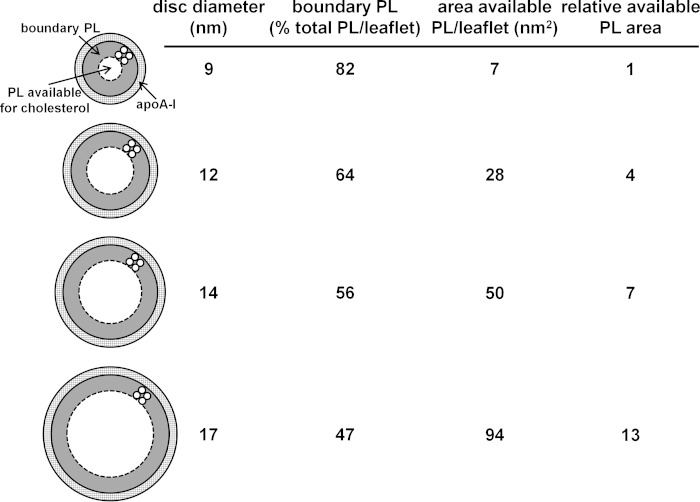
**Model showing the influence of molecular packing constraints in discoidal HDL particles of different sizes on cholesterol-solubilizing capacity.** The molecular packing of the PL molecules adjacent to the α-helices of the apoA-I molecules wrapped around the circumference of the discs is constrained so that their ability to solvate cholesterol molecules is reduced (see text for further details). The diagram shows a top down view of four HDL discs with their diameters drawn to scale. The apoA-I molecules are assumed to be arranged as a double belt (61) with closely packed α-helices occupying 0.15 nm^2^/amino acid residue (62). The thickness of the cylindrical cross-section formed by such helices is 1 nm. The boundary layer of PL adjacent to the annulus of apoA-I is assumed to be two molecules wide (52). The PL is assumed to occupy a molecular area of 0.65 nm^2^ (63), which corresponds to a circular cross-section of ∼1-nm diameter. The major diameter of the disc-shaped particle is assumed to be similar to the hydrodynamic diameter (55, 64). The areas of each bilayer leaflet occupied by boundary PL and the remaining PL (labeled as PL available for cholesterol) are calculated for the 9, 12, 14, and 17 nm HDL discs using the above dimensions. The fraction of PL forming the boundary layer varies inversely with diameter for the discoidal HDL particles so that the fractional availability of PL for solvating cholesterol is low in small HDL particles. It is apparent that approximately doubling the particle diameter from 9 to 17 nm leads to a 13-fold increase in the amount of PL into which cholesterol can dissolve (relative available PL area). As a consequence, cholesterol preferentially distributes to larger nascent HDL particles during formation by membrane solubilization.

The relative FC enrichment in larger nascent and reconstituted HDL particles (Figs. 1, 5, and 6 and Table 1) is a result of particle size-dependent constraints on the packing of PL molecules. It is well established that the packing of PL molecules immediately adjacent to the annulus of apoA-I molecules at the disc circumference is affected so that FC molecules do not dissolve well in this “boundary layer” (16, 45–47, 50–52). Fig. 10 summarizes how the proportion of PL molecules located in the boundary layer varies with disc size. It is apparent from this model that the cholesterol-solubilizing capacity can vary by an order of magnitude for discs in the 9–17-nm range. The small fraction of PL available to accommodate dissolved cholesterol molecules in discs with diameters of 9 nm explains qualitatively the reductions in FC/PC ratio with decreasing disc size shown in Fig. 5. The ratio (available PL area)/(total PL area) is presumed to predict the measured FC/PL ratio for a given disc size. The model predicts for the 14 and 12 nm FC-containing DMPC discs that this ratio has values of 0.44 and 0.36, respectively, corresponding to a relative FC/DMPC content of 1.2/1 for the large and small discs. The measured ratio for the 14 nm and 12 nm discs is 0.04/0.03 = 1.3/1 (*lower panel* in Fig. 6), indicating that the theoretical model provides a reasonable fit. In the case of HDL discs formed by solubilization of membrane-PL MLV, similar calculations for 12- and 9-nm discs predict a relative FC/PC (w/w) ratio of 2/1, whereas the experimentally observed value for discs of these sizes is 0.06/0.02 = 3:1 (Fig. 5, *lower panel*). The 11- and 8-nm nascent HDL discs originating from BHK-ABCA1 cells also have relative FC/PL contents that differ by a factor of 3 (Table 1). The HDL discs derived from the BHK cell plasma membrane (containing ∼30 mol % FC relative to PL) are commensurately enriched in FC relative to the model MLV systems of Figs. 5 and 6 but, importantly, the PL boundary layer effects on FC content (Fig. 10) still apply. It is interesting that in cell-free systems although metastable PC vesicles containing more than equimolar levels of FC can be created (53), reconstituted discoidal HDL particles containing more than ∼30 mol % FC do not form (44, 45, 54, 55). At this stage, the reasons for this difference between the cell-free and cell systems are not entirely clear, although it is known that the presence of 40 mol % FC in PL vesicles can enhance apoA-I binding (56) so it is unsurprising that apoA-I can bind to vesiculated domains formed in the plasma membrane of BHK cells by ABCA1 activity. The ongoing lipid-translocase activity of the transporter may provide additional bilayer destabilization allowing FC-rich nascent HDL particles to form. It is noteworthy that the relative exclusion of FC from the smaller HDL discs (Fig. 10) can lead to FC enrichment of larger discs to levels greater than that of the FC/PL bilayer being solubilized. It follows that the occurrence of high FC/PL levels in nascent HDL particles is not necessarily a consequence of FC-rich plasma membrane raft domains being solubilized.

The experimental results summarized in Fig. 7 indicate that discoidal HDL particles of different sizes can accommodate PC and SM similarly, implying that boundary layer effects (Fig. 10) are the same for both classes of PL. The variations in the relative amounts of PC and SM observed with nascent HDL can apparently be accommodated readily within the disc structure. In contrast, the accommodation of PS is sensitive to disc size, with smaller discs being preferred (Fig. 8). This result is consistent with PS packing better than PC in the boundary layer (Fig. 10). This effect may arise because electrostatic repulsion between the negatively charged polar groups causes PS to form more highly curved interfaces with water than PC does (57) so that the edge of small highly curved discs is preferred. This location for PS may be further enhanced by PS-apoA-I interactions because it is known that the presence of PS enhances apoA-I binding to an SUV surface (6). Any such accumulation of PS in small discoidal HDL particles would be expected to increase the net negative particle charge thereby giving rise to α-electrophoretic mobility (58).

##### Summary and Conclusions

The detergent-like ability of apoA-I to penetrate into and solubilize PL/FC bilayer membranes under appropriate conditions (59, 60) underlies the process by which the heterogeneity of nascent HDL arises. Nascent HDL does not have a unique composition; this parameter is dependent on cell type and status, and the relative availability of apoA-I. Different sizes of discoidal HDL particles are created concurrently during the solubilization reaction with larger particles having a higher FC/PL ratio. The relative depletion of FC molecules in smaller discs is due to the following: (*a*) a larger fraction of the PL forming a boundary layer adjacent to the apoA-I molecules located around the disc edge and (*b*) the boundary layer PL being unable to solvate FC molecules appropriately. Thus, molecular packing constraints in the nanoscale HDL discs, rather than the location of ABCA1 in membrane microenvironments with different FC contents, are responsible for heterogeneity of FC content in nascent HDL subspecies.

Knowledge of the molecular basis for the origins of HDL heterogeneity should help in the development of methods for enhancing formation of subspecies with desired functionalities. For instance, different types of HDL particles exhibit varying abilities to support reverse cholesterol transport and exert different levels of cardio-protection (1, 2). Small HDL particles, which are low in FC, are reported to display potent anti-atherogenic properties (2) so selecting for such particles could be beneficial.

## Footnotes

2 The abbreviations used are:

apo: apolipoprotein
ABCA1: ATP binding cassette transporter A1
BHK: baby hamster kidney
DMPC: dimyristoyl phosphatidylcholine
DPPC: dipalmitoyl phosphatidylcholine
FC: free (unesterified) cholesterol
MLV: multilamellar vesicles
PC: phosphatidylcholine
PS: phosphatidylserine
PL: phospholipid
SM: sphingomyelin
SUV: small unilamellar vesicle(s).
